# Endovascular reconstruction of bilateral upper limbs ischemia in a patient with arterial outlet syndrome: A case report and literature review

**DOI:** 10.3389/fsurg.2022.951956

**Published:** 2022-09-07

**Authors:** Mi Zhou, Wei Jia, Peng Jiang, Zhiyuan Cheng, Yunxin Zhang, Jianlong Liu

**Affiliations:** Department of Vascular Surgery, Beijing Jishuitan Hospital, Beijing, China

**Keywords:** arterial thoracic outlet syndrome, endovascular, ischemia, Rotarex, drug-coated balloon

## Abstract

**Background:**

Acute upper limb ischemia in a patient with thoracic outlet syndrome is a rare but serious clinical disorder. If the disease is not treated promptly due to underdiagnosis, it could lead to distal artery embolization and limb-threatening ischemia. Revascularizing upper extremity arteries in a timely manner could rescue ischemic limbs and improve the patient’s quality of life. We reported here a case of a patient who presented with bilateral upper limb ischemia caused by arterial thoracic outlet syndrome.

**Case presentation:**

A 63-year-old woman who presented with sudden bilateral upper extremity cold, numbness, pulselessness, and altered temperature sensation was first diagnosed with arterial thoracic outlet syndrome. The patient had performed a lot of pull-up and lat pull-down exercises in the 2 months prior to the onset of the above symptoms. Color Doppler ultrasonography showed thrombosis in the right axillary artery and left subclavian and axillary artery. The patient received Rotarex mechanical thrombectomy combined with drug-coated balloon percutaneous transluminal angioplasty (PTA) to complete revascularization of the upper extremities and achieved a full recovery finally.

**Conclusions:**

Complete endovascular revascularization for treating arterial thoracic outlet syndrome is a minimally invasive and effective method, especially for upper extremity ischemic lesions caused by nonbone compression.

## Background

Arterial thoracic outlet syndrome (TOS) is a group of disorders manifested with upper extremity ischemia or aneurysmlike disease caused by external compression of subclavian or axillary arteries at the thoracic outlet (1). Arterial TOS is the least common type of thoracic outlet syndrome, accounting for only 1%–2% of all TOS cases (2–5). The principal anatomic pathology structures include abnormal development of anterior scalene muscle, hypertrophy cervical rib, and anomalous first rib, which leads to aneurysm or occlusion of subclavian or axillary arteries (6).

Decompression of the thoracic outlet is the classic therapy, including cervical rib excision, release of scalenus, and vascular thrombectomy and reconstruction (7). However, the above surgery types cause great trauma, heavy bleeding, and many complications (8–10); in addition, the constriction of scar tissues at the incision site may cause recurring symptoms (4, 11). The advancement of endovascular technology and the innovation of interventional devices make endovascular therapy possible to cure a considerable number of diseases that required open surgery treatment in the past.

We report a rare case of acute ischemia of bilateral upper extremities caused by arterial thoracic outlet syndrome. Rotarex mechanical thrombectomy combined with a drug-coated balloon was used to reconstruct the upper extremity blood flow, and finally, the patient fully recovered.

## Case presentation

**Figure 1:**
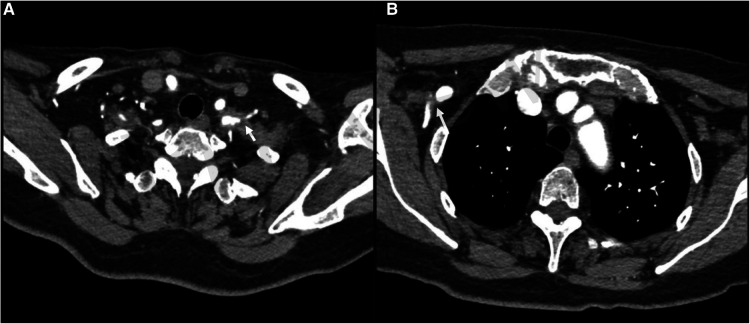
Preoperative CT scan revealing soft tissue compression of the right axillary artery (**B**) and the left subclavian and axillary artery (**A**).

A 63-year-old female patient presented with acute bilateral upper extremity cold, pulselessness, and altered temperature sensation. The patient described that she had performed a lot of pull-up and lat pull-down exercises in the 2 months prior to the onset of the above symptoms. The physical examination showed that typically marked bilateral upper extremity ischemia manifesting with radial and ulnar artery blood flow were out of palpable and cold sensation in upper limbs. The patient was diagnosed with arterial thoracic outlet syndrome after clinical provocation tests (Wright test, Roos test, Adson test) and computed tomography (CT) (Figure 1) results showing artery compression at the thoracic outlet. Doppler ultrasonography showed thrombosis in the right axillary artery and left axillary and subclavian artery. The patient did not have a history of atrial fibrillation, and echocardiography did not suggest intra-atrial thrombus. In addition, further laboratory test results (Table 1), especially immunological tests and thrombophilia-related tests, basically excluded the possibility of upper extremity ischemia due to atherosclerotic occlusion or vasculitic disorders.

**Table 1:** Overall laboratory results (complete blood count, blood chemistry, coagulation function test, and immunological tests) of the patient.

Component measured	Reference range	Result
Hemoglobin (g/L)	105–140	116
Platelet count (×10^9^/L)	125–350	468
Cholesterol (mmol/L)	<5.18	5.37
Triglycerides (mmol/L)	<1.7	0.96
CRP (mg/L)	0–8	41.7
D-dimer (mg/L)	0–0.5	8.3
FIB (mg/dl)	200–400	534
PC (%)	70–140	121
PS (%)	63.5–149	106.7
AT (%)	83–128	107
VIII (%)	50–150	167.1
C3 (g/L)	0.79–1.52	1.45
C4 (g/L)	0.16–0.38	0.4
ESR (mm)	39	0–20

CRP, C-reactive protein; FIB, fibrinogen, PC, protein C; PS, protein S; AT, antithrombin; VIII, VIII factor; ESR, erythrocyte sedimentation rate.

**Figure 2:**
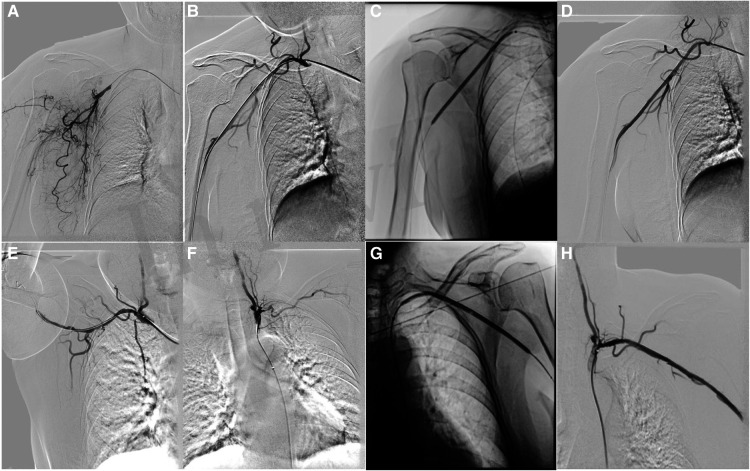
Rotarex thrombectomy combined with drug-coated balloon dilation in treating arterial TOS. (**A**) Baseline angiography showing right axillary artery occlusion. (**B**) Angiography after plain old balloon angioplasty in the right axillary artery. (**C**) Drug-coated balloon (DCB) angioplasty in the axillary artery. (**D**) Angiography after DCB angioplasty in the right axillary artery. (**E**) Angiography of the right axillary artery in the excessive abducted and hyperextension position. (**F**) Baseline angiography showing left subclavian and axillary artery occlusion. (**G**) Angiography after DCB angioplasty in the left subclavian and axillary artery. (**H**) Angiography after DCB angioplasty in the left subclavian and axillary artery.

The patient received anticoagulant and antiplatelet therapy after hospitalization. An 8F Rotarex (Straub, Switzerland) mechanical thrombectomy system was used to reduce the thrombus burden in the axillary artery and the subclavian artery. To decrease the risk of ectopic embolization, we adopted a strategy of slowly pushing the Rotarex catheter from proximal to distal end of the artery. Before the aspiration of thrombus in the distal artery, we performed repeated aspiration in the proximal artery aiming to reduce the amount of thrombus fully. Then, 3-, 5-, and 8-mm Mustang balloon catheters (Bosten Scientific, United States) (Figures 2C,G) were used to dilate the target vessels. An 8-mm Acotec drug-coated balloon catheter (Xianruida, China) was implanted subsequently to prevent neointimal hyperplasia and recurrence of thrombosis. Angiography shows that residual stenosis is less than 30% (Figures 2D,H) when the upper extremity is in the excessive abduction and hyperextension position (Figure 2E). The patient received strict anticoagulation (rivaroxaban, 20 mg/day) and antiplatelet (aspirin, 100 mg/day) therapy postoperation for 6 months and achieved full recovery. During the 6-month follow-up, bilateral upper extremity arteries were patent, with no recurrence of symptoms.

## Discussion

Arterial thoracic outlet syndrome is usually present with upper extremity ischemia or aneurysm caused by chronic external compression of the subclavian artery or axillary artery when passing through the defined anatomical space called thoracic outlet. Bony abnormalities such as abnormal cervical rib and first rib, anomalous development of anterior scalene muscle, and other soft tissues contribute to subclavian/axillary artery stenosis, occlusion, thrombosis, and aneurysm. The innovation of endovascular technology makes it possible to treat arterial thoracic outlet syndrome through a more minimally invasive surgical approach.

First, the diagnosis of arterial TOS needs to be carefully distinguished. Patients with arterial TOS usually present with obvious symptoms of limb ischemia such as cold feeling, pulseless, and altered temperature sensation. In addition to pain, numbness, and paresthesia, some neurogenic TOS patients also manifested intermittent finger paresthesia, discoloration, and coldness in the absence of thromboembolism in the subclavian artery. The ischemic manifestations of the upper extremities are mainly attributed to vasospasm mediated by sympathetic nerve plexus compression rather than true arterial TOS (12, 13). Color Doppler ultrasonography (14), computed tomography angiography (CTA) (15), magnetic resonance angiography (MRA) (16), and digital subtraction angiography (DSA) are indispensable to distinguish arterial TOS from neurological TOS (17). At the same time, arterial TOS and neurogenic TOS may coexist in some patients who presented with overlapping symptoms. In addition, subclavian/axillary artery stenosis or thrombosis caused by arterial TOS is needed to be distinguished from arteriosclerotic and vasculitic subclavian artery stenosis. Arteriosclerosis is the most common cause of subclavian artery stenosis in elderly patients, and the target lesions are often limited to the proximal part of the subclavian artery, rarely involving the distal subclavian artery beyond the opening of the vertebral artery and axillary arteries. Vasculitic subclavian artery stenosis is often accompanied by markedly elevated erythrocyte sedimentation rate (ESR) and C-reactive protein (CRP) and is usually combined with carotid artery stenosis/occlusion. Obvious vascular compression signs can be founded in CTA scans, and various provocative tests (Wright test, Roos test, Adson test, Eden test) (18) are also helpful to differentiate arterial TOS from arteriosclerotic and vasculitic subclavian stenosis.

The congenital abnormality of anterior scalene muscle, cervical rib, the first rib, and other factors lead to the compression of the subclavian/axillary artery, resulting in intimal hyperplasia, thrombosis, and aneurysm in arterial TOS. Traditional surgery requires thoracic outlet decompression, artery release, thrombectomy, and subclavian–axillary artery bypass grafting, causing extremely traumatic injury and bleeding. The advancement of endovascular intervention provides new treatment possibilities. Pantoja et al. (19) compared open surgery with endovascular therapy for treating arterial TOS and found that endovascular treatment had shorter operation time, lesser blood loss, and shorter hospitalization. In addition, there was no statistical difference in primary patency, assisted primary patency, and secondary patency between the open group and the endovascular group. In addition, the patient’s pain score and QuickDASH (arm, shoulder, and hand disability) score decreased significantly after surgery. Previous studies showed good clinical efficacy of endovascular treatment, which is consistent with our findings, indicating the advantages of endovascular interventional techniques in treating arterial TOS. A recent systematic review by Maskanakis et al. (20) included 73 studies and analyzed subclavian artery stenting in patients with arterial TOS and found that the technical success rate was 80%–100%, and the reintervention rate was 9%. However, there were some problems with stent implantation in treating arterial TOS. Archie et al. (21) summarized the clinical efficacy of subclavian artery stent implantation in seven arterial TOS cases manifested by aneurysms. Two patients received catheter thrombolysis due to in-stent thrombosis within 3 years after surgery, and one patient underwent drug-coated balloon dilation for in-stent restenosis in the second year after surgery. During the diagnosis of our patient, CT results suggested that the bilateral upper extremity artery occlusion was caused by compression of soft tissues, other than bony compression. The reasons why we did not manage anatomical abnormalities in this patient are as follows: the patient had an absolutely incentive cause for the onset of the disease, and she did not have the typical symptoms of TOS such as obvious upper extremity ischemia and numbness in the past, indicating that the patient’s initial compression of the thoracic outlet is not very serious. Excessive hyperextension and abduction exercises aggravated soft tissue compression of the artery, resulting in intimal injury and thrombosis. Therefore, we instructed the patient to pay more attention to performing conservative treatment, including modification of behavior patterns and postural correction after endovascular reconstruction of bilateral upper limbs’ blood flow. Many previous studies (22–26) have also found that conservative treatment should be the initial treatment for TOS except in cases of acute thromboembolism and progressive neurological insufficiency (25, 27, 28). Whether the surgery treatment is necessary for TOS is still controversial (29). We suggest that the choice of therapy needs to be individualized. The main reasons for adopting the Rotarex thrombectomy device combined with a drug-coated balloon to treat arterial TOS in our case are as follows: (1) the Rotarex thrombectomy device can reduce the intraluminal thrombosis burden to the greatest extent and prevent distal ectopic artery embolism. Intracranial arterial embolism caused by arterial TOS has been reported in previous studies (30, 31), so we pushed the Rotarex device from the proximal to the distal end of the artery in the direction of antegrade blood flow during the thrombectomy process, especially involving the vertebral artery lesion. (2) We carried out drug-coated balloon dilation to prevent intimal hyperplasia and arterial restenosis during arterial remodeling, which affects the long-term patency rate; meanwhile, it avoids the possibility of thrombosis, restenosis, and fracture caused by stent implantation.

Rotarex thrombectomy combined with drug-coated balloon dilation achieved good initial clinical results and a satisfactory arterial patency rate in treating arterial TOS; however, a strict case screening is required. For arterial TOS caused by bone compression, it is still necessary to decompress the neurovascular bundle first (32). Further close follow-up after endovascular reconstruction is indispensable for arterial TOS.

## Data Availability

The original contributions presented in the study are included in the article/Supplementary Material, further inquiries can be directed to the corresponding author.
